# Generation of a medicine food homology formula and its likely mechanism in treatment of microvascular angina

**DOI:** 10.3389/fphar.2024.1404874

**Published:** 2024-08-30

**Authors:** Zhidie Jin, Mingwang Liu, Beili Xie, Wei Wen, Yuxin Yan, Yangfang Zhang, Haohao Li, ZhengYu Shen, Lulian Jiang, Mengjie Gao, Keji Chen, Fuhai Zhao

**Affiliations:** ^1^ Xiyuan Hospital, China Academy of Chinese Medical Sciences, Beijing, China; ^2^ Graduate School of Beijing University of Chinese Medicine, Beijing, China; ^3^ Affiliated Hospital of Shanxi University of Traditional Chinese Medicine, Shanxi University of Traditional Chinese Medicine, Taiyuan, China

**Keywords:** medicine food homology, data mining, network pharmacology, molecular docking, east asian traditional medicine, microvascular angina, coronary microvascular disease, coronary artery disease

## Abstract

Microvascular angina (MVA) is the most common cause of cardiac ischemic chest pain in patients without obstructive coronary artery disease (CAD) and lacks of effective treatment means. Medicine food homology (MFH) involves substances with both nutritional and medicinal qualities that have the potential to improve MVA symptoms as medicines, dietary supplements. However, research on MFH formula (MFHF) for MVA is not available. The study aims to generate a core MFHF for MVA through data mining and offer scientific backing for the utilization of edible medications in the prevention and alleviation of MVA. 11 databases were utilized to construct a database of MFH drugs, and the MFHF was generated through frequency analysis, association rule analysis, and clustering analysis. The composition of the formula is Codonopsis Radix, Astragali Radix, Platycodonis Radix, Persicae Semen, Glycyrrhizae Radix Et Rhizoma, Angelicae Sinensis Radix, and Allii Macrostemonis Bulbus. Through network pharmacology and molecular docking, we identified five major active components of MFHF: Adenosine, Nonanoic Acid, Lauric Acid, Caprylic Acid, and Enanthic Acid, along with nine core targets (NFKB1, ALB, AKT1, ACTB, TNF, IL6, ESR1, CASP3, and PTGS) for the improvement of MVA. These 5 active components have various biological activities, such as reducing oxidative stress, anti-inflammation, analgesia effect, inhibiting platelet aggregation, vasodilatation, vascular endothelial protection, and cardio-protection. GO and KEGG enrichment analyses revealed that MFHF mainly acted on the response to xenobiotic stimulus, integrative component of the plasma membrane, RNA polymerase II transcription factor activity, ligand-activated sequence-specific DNA binding, pathways in cancer, lipid and atherosclerosis, human cytomegalovirus infection, and the PI3K-Akt signaling pathway, which are the main pathogenesis of MVA.

## 1 Introduction

Microvascular angina (MVA) falls under the umbrella of ischemia and non-obstructive coronary artery disease (INOCA) Ang et al. (2022). Nearly 72% of INOCA patients exhibit coronary microvascular dysfunction, among whom 52% specifically present with isolated primary MVA (Al-Mohaissen, 2023). As a type of coronary artery disease (CAD), MVA has manifestations of coronary microvascular dysfunction (CMD) and usually refers to coronary microvascular disease (CMVD) without obstructive coronary artery disease, but its concept may extend further to include patients with obstructive coronary artery disease and after coronary revascularization or heart transplantation (Berry et al., 2022; Abouzid et al., 2024). The potential for major adverse cardiovascular events, like cardiovascular death, is increased by MVA. Besides, MVA is also associated with a poor prognosis and a poor quality of life (Zhong et al., 2020). Patients with CMVD remain under-treated because existing management guidelines do not address this large, mostly female population due to the absence of evidence-based data. Currently, western treatment for patients with CMVD centres on symptom and primary risk factor therapy. Common treatments like beta-blockers, statins, ACEIs/ARBs, and CCBs lack extensive clinical trial data on efficacy and safety (Soleymani et al., 2022). Non-pharmacological treatments, including transcutaneous neurostimulation, and cognitive behavioral therapy, lack strong evidence-based recommendations (Liang et al., 2020). Therefore, it is crucial to focus on preventive and therapeutic approaches that target the underlying disease mechanisms of MVA to reduce associated morbidity and mortality risks.

The advantages of Traditional Chinese Medicine (TCM) in the treatment of MVA have been confirmed by many clinical studies (Yang et al., 2022). However, although TCM is efficacious, it still has serious side effects when used incorrectly (Hou and Jiang, 2013). Foods have less prominent and rapid therapeutic effects, but they have milder side effects, can be taken safely at higher doses, and can be taken for longer periods of time (Hou and Jiang, 2013). Both come from the same source, and there is no absolute boundary between the two. Medicine food homology (MFH) refers to substances traditionally used as food and listed in the Chinese Pharmacopoeia. National Health Commision of the People’s Republic of China has recently announced 110 MFH drugs (Qu et al., 2023). MFH scientifically combines the functions of food and medicine by incorporating nutritional value, healthcare activities, and disease prevention and alleviation (Chien et al., 2015). With the growing global trend towards returning to nature and simplicity, there is a rising demand for MFH not only in China but also worldwide (Qu et al., 2023). The MFH formula (MFHF) can combine the advantages of MFH with TCM compound prescriptions. However, there are no studies on MFH for the treatment of MVA, and the specific mechanism of which is still unclear. These limitations hinder the application of MFH drugs in the prevention and alleviation of MVA. Therefore, it is urgently needed to develop new drugs.

The current study utilized data mining techniques to construct a fundamental formula for the treatment of MVA from MFH drugs. The underlying pharmacodynamic components and possible molecular mechanisms were also explored through the network pharmacology of this MFHF. Simultaneously, the main active ingredients were docked with core targets for preliminary validation.

Data mining is an important tool for discovering relationships and potential associations between massive amounts of data (Liu et al., 2023). Some mining methods have been very successful in studying the combinatorial patterns of traditional Chinese medicines (Liu et al., 2023).

Network pharmacology, a burgeoning field, integrates experimental, clinical, and computational approaches to enhance drug effectiveness (Sadaqat et al., 2023). This innovative trend has found applications across diverse domains, including cardiovascular diseases, paving the way for improved therapeutic outcomes (Sadaqat et al., 2023).

Molecular docking is the utilization of computer technology to simulate the geometric structure and spatial interactions between drug ingredients and disease targets in order to identify ingredients with therapeutic significance.

This strategy provides a new way to fully utilize the existing food and drug resources to promote the development of MVA prevention and symptomatic alleviation.

**FIGURE 1:**
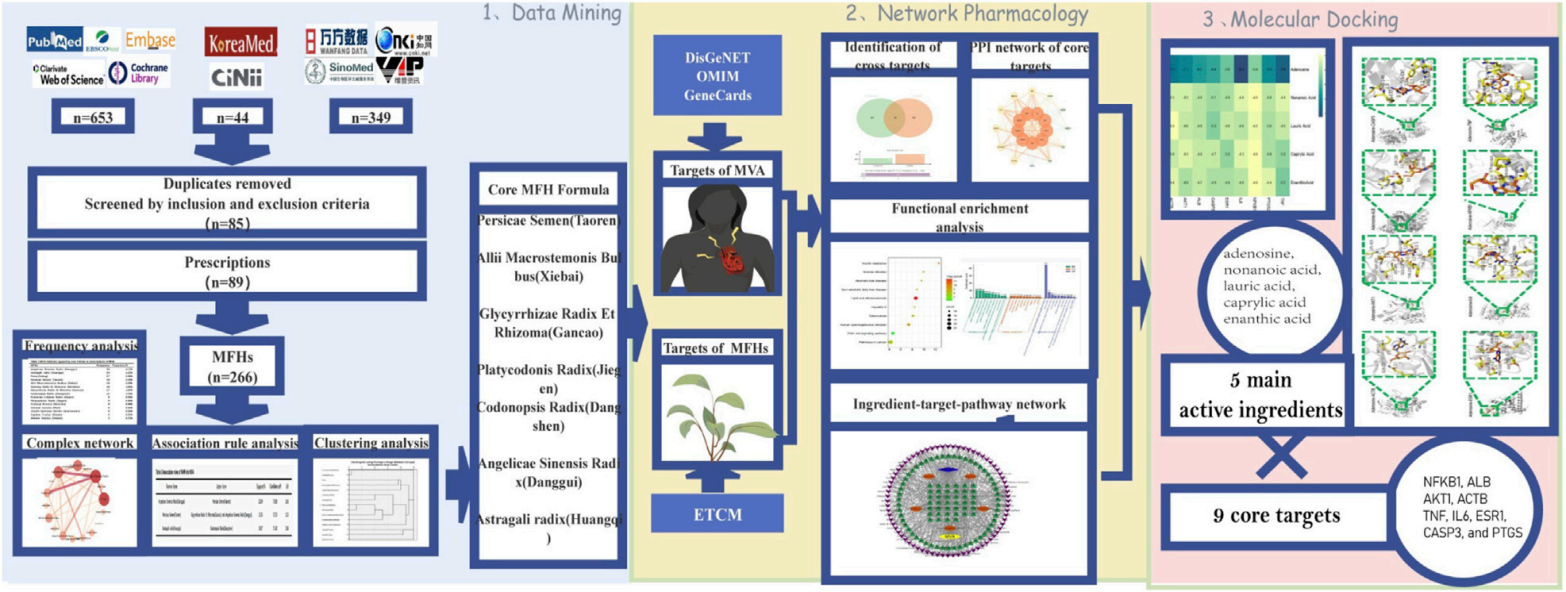
Research methodology flowchart.

The overall flow chart of this study is shown in Figure 1.

## 2 Materials and methods

### 2.1 Data mining

#### 2.1.1 Data sources and screening

Historically, MVA was called CSX, numerous investigations on coronary microvascular dysfunction in CSX patients support MVA. So we included CSX-related keywords.

East Asian traditional medicine (EATM) encompasses several traditional medical practices, such as traditional Chinese medicine (TCM), traditional Mongolian Medicine (TMM), traditional Tibetan medicine (TTM), Kampo Medicine, and traditional Korean medicine (TKM), among others. While each of EATM has unique attributes, they share academic, medical, and medicinal expertise, and some of them have developed from TCM, which is rooted in the theory of the MFH (Qu et al., 2023). So, some of these EATMs may include MFH drugs.

Therefore, we searched for TCM prescriptions for MVA through 4 Chinese databases (CNKI, WanFang Data, VIP, CBM) and 5 English databases (PubMed, EBSCOhost, The Cochrane Library, Embase, Web of Science) for EATM prescriptions. We also searched Japanese CiNii for Kampo Medicine and Korean database KoreaMed for TKM prescriptions.

After examining CBM and MeSH subject and free terms, the final search terms were determined. 1 January 2000, to 25 October 2023, is the search timeframe. The retrieval methodology and keywords are detailed in Table 1. The database was screened for relevant articles using Endnote.

**TABLE 1:** Retrieval methodology for documents on prescriptions in microvascular angina.

Retrieval methodology	Keywords content
A. Retrieval methodology to locate “microvascular angina”	“Angina, Microvascular”, “X Syndrome, Angina”, “Angina X Syndrome”, “Angina X Syndromes”, “Syndrome, Angina X″, “Syndrome X, Cardiac”, “Syndrome X, Angina”, “Angina Syndrome X”, “Angina Syndrome Xs”, “Syndrome Xs”, Angina”, “Angina Pectoris with Normal Coronary Arteriogram”, “Cardiac Syndrome X”
B. Retrieval methodology to locate “Traditional East Asian Medicine”	“Traditional East Asian Medicine”, “Medicine, Traditional, East Asia”, “Traditional Medicine, East Asia”, “Traditional Far Eastern Medicine”, “East Asian Traditional Medicine”, “Oriental Traditional Medicine”, “Medicine, Oriental Traditional”, “East Asian Medicine”, “East Asian Medicines”, “Medicine, East Asian”, “Oriental Medicine”, “Medicine, Far East”, “East Medicine, Far”, “East Medicines, Far”, “Far East Medicine”, “Far East Medicines”, “Medicines, Far East”, “Medicine, East Asia”, “Asia Medicines, East”, “East Asia Medicine”, “East Asia Medicines”, “Medicines, East Asia”, “Medicine, Oriental”
C. Retrieval methodology to locate “Medicine, Chinese Traditional”	“Medicine, Chinese Traditional”, “Traditional Chinese Medicine”, “Chung I Hsueh”, “Hsueh, Chung I”, “Traditional Medicine, Chinese”, “Zhong Yi Xue”, “Chinese Traditional Medicine”, “Chinese Medicine, Traditional”, “Drugs, Chinese Herbal”, “Chinese Drugs, Plant”, “Chinese Herbal Drugs”
C. Retrieval methodology to locate “Medicine, Chinese Traditional”	“Herbal Drugs, Chinese”, “Plant Extracts, Chinese”, “Chinese Plant Extracts”, “Extracts, Chinese Plant”, “Herbal Therapy”, “Herb Therapy”, “Herbal Medicine”
D. Retrieval methodology to locate “Medicine, Mongolian Traditional”	“Medicine, Mongolian Traditional”, “Medicines, Mongolian Traditional”, “Mongolian Traditional Medicine”, “Mongolian Traditional Medicines”, “Traditional Medicine, Mongolian”, “Traditional Medicines, Mongolian”, “Mongolian Medicine”, “Medicine, Mongolian”, “Medicines, Mongolian”, “Mongolian Medicines”, “Mongolian Folk Medicine”, “Folk Medicine, Mongolian”, “Folk Medicines, Mongolian”, “Medicine, Mongolian Folk”, “Medicines, Mongolian Folk”, “Mongolian Folk Medicines”
E. Retrieval methodology to locate “Medicine, Tibetan Traditional”	“Medicine, Tibetan Traditional”, “Tibetan Traditional Medicine”, “Tibetan Medicine, Traditional”, “Medicine, Traditional Tibetan”, “Traditional Tibetan Medicine”, “Tibetan Medicine”, “Medicine, Tibetan”, “Traditional Medicine, Tibetan”
F. Retrieval methodology to locate “Medicine, Korean Traditional”	“Medicine, Korean Traditional”, “Traditional Medicine, Korean”, “Traditional Medicine, Korea”, “Korea Traditional Medicine”, “Medicine, Korea Traditional”, “Korean Traditional Medicine”, “Sasang Constitutional Medicine”, “Medicine, Sasang Constitutional”
J. Retrieval methodology to locate “Medicine, Kampo”	“Medicine, Kampo”, “Kanpo Medicine”, “Medicine, Kanpo”, “Kanpo”, “Kampo”, “Kampo Medicine”

The inclusion criteria for screening data were: (1) Types of studies: randomized controlled trials, observational studies, experience of famous doctors, medical records, case reports. (2) The included patients met the diagnostic criteria for MVA in Western medicine. Patients’ gender, age, and region were not restricted. (3) Intervention: Oral intestinally absorbed compounded medications, including patented Chinese medicines, granules, decoctions, pills, powders, capsules, and other internal therapies, without dosage or duration limits. (4) Complete and thorough prescription, including drug composition or approval number for traceability. (5) If duplicate prescriptions were found in different literature, only the most clinically complete was included.

The exclusion criteria for screening data were: (1) Reviews, conferences, guidelines, expert consensus, theoretical discussions, basic experimental investigations, pharmacological studies, and database algorithm-based literature for formula development or drug summaries. (2) Diagnostic inconsistency. (3) Combined with complex and major diseases. (4) Nonprescription literature or studies that used prescription therapy only as a control group; studies that did not demonstrate superior outcomes with prescription therapy; single-drug prescriptions; non-oral trans-intestinal absorption, like acupuncture, injections, sprays, and other external therapies.

To ensure data accuracy and dependability, two researchers independently screened and extracted literature, double-checked data, and resolved disagreements.

#### 2.1.2 Data standardization and database establishment

A prescription database was created using formulas that met the inclusion and exclusion criteria in Microsoft Excel 2010. The database included information such as literature name, year, author, prescription name, composition, and code.

The drug names were standardized according to the 2020 Pharmacopoeia of the People’s Republic of China. All processed products were ignored unless the Pharmacopoeia specified them individually. Here, “stir-fying Maiya” is standardized as “Maiya” and “stir-fying Zaoren” as “Suanzaoren.” Due to challenges in standardizing and regulating herbal medicine dosages, we do not account for dosage variations.

Record screening and standardization in the database. Standardized medicines were entered into Excel 2010 to construct a drug database. We searched the National Health Commission of the People’s Republic of China (http://www.nhc.gov.cn/wjw/index.shtml) and the China Quality News Network (https://www.cqn.com.cn/) for the MFH list. A new MFH database was created from the drug database.

A matrix of medicinal drugs was created in Excel 2010. The data was formatted into Y and N. The library can be imported into IBM SPSS Modeler 18.0 for association rule analysis and complex network analysis. Edit the data to convert it into a format of 1s and 0s. The library can be imported into SPSS 20.0 for cluster analysis.

#### 2.1.3 Data analysis and genreation of a core MFH formula

Frequency analysis: Frequency statistics of the MFH drugs were analyzed using Microsoft Excel 2010 software. The frequency and percentage of MFH drugs occurrence were calculated. High-frequency drugs (>4 occurrences) were screened.

Association analysis: the Apriori algorithm in IBM SPSS Modeler 18.0 was utilized to analyze the association rules of MFH drugs. The conditions set were a minimum support at 10% and a minimum confidence of at 70% to extract drug pair rules and identify potential associations between them. Association rules are evaluated based on their support, confidence, and lift.

SPSS Modeler 18.0 is used for complex network analysis, with specific settings for display of true flags, maximum links, and strong links. The network consists of 38 nodes and 171 edges, with black edges representing interactions. Cytoscape 3.9.1 is used to visualize the network and determine node significance. Topological parameters like degree, betweenness centrality, and closeness centrality are calculated to determine the importance of nodes. Higher values indicate greater importance. After filtering data based on degree, 18 nodes and 97 edges are obtained to construct the complex network of MFH drugs.

Hierarchical Cluster Analysis: Cluster analysis can determine drug compounding patterns and identify prescription combination rules (Liu et al., 2023). We utilized IBM SPSS Statistics 25 to perform a hierarchical cluster analysis on high-frequency MFH drugs, using the cluster method “between-groups linkage,” “Euclidean distance,” and “Jaccard binary.” The results are presented in a dendrogram.

These statistical analysis methods were employed to identify the seven core MFH drugs in the formula, which are not only compatible with prescription combination rules and commonly used but also have to target TCM patterns for MVA perspectively.

### 2.2 Network pharmacology

#### 2.2.1 Active ingredient screening and target prediction

Combining the results of frequency statistics, association rules, complicated networks, and systematic cluster analysis, we discovered core MFH drugs and developed a novel formula.

The Encyclopedia of Traditional Chinese Medicine database (ETCM, http://www.tcmip.cn/ETCM/) was used to obtain the core MFH formula ingredients and candidate target genes. MedChem Studio (version 3.0; Simulations Plus, Inc., Lancaster, CA, United States, 2012) provided, ETCM’s drug target gene data. In the, ETCM database, input ingredients with a Tanimoto >0.8 can be searched for drug targets by utilizing MedChem Studio, a powerful tool for drug-like property searches. Drug-likeness evaluations of ingredients were based on a quantitative estimate model by the Bickerton group (Xu et al. (2019). Quantitative estimates of drug-likeness (QED) scores >0.49 were used to screen active ingredients. Active ingredients having QED scores <0.49 but listed in the 2020 Pharmacopoeia were also considered.

#### 2.2.2 Collection of MVA-related targets

We Uutilized the GeneCards database (https://www.genecards.org/), the OMIM database (https://www.omim.org/), and the Disgenet database (https://www.disgenet.org/) to do a search using the term “microvascular angina.” After retrieving data from the GeneCards database, we employed the “relevance score” to find disease targets that ranked higher than the median. The targets were merged with two other datasets to eliminate duplicates and generate the final targets connected to MVA.

#### 2.2.3 Obtaining cross target genes of MFHs and MVA

The bioinformatics platform (https://www.bioinformatics.com.cn/) was utilized to create cross-targets and Venn diagrams of MFHs and MVA. These cross-targets are potential targets for preventing and treating MVA with MFHs.

#### 2.2.4 GO and KEGG enrichment

The target gene list was enriched with cross-targets using the DAVID database (https://david.ncifcrf.gov/), specifying “*Homo sapiens*” as the species. Predicted gene functions were assessed through Gene Ontology (GO) and Kyoto Encyclopedia of Genes and Genomes (KEGG) enrichment analyses. GO functions include biological process (BP), molecular function (MF), and cellular component (CC). The top 10 GO terms and KEGG pathways with *p*-values <0.01 and higher counts were visualized through a bioinformatics platform (https://www.bioinformatics.com.cn/) using histogram and bubble diagram.

#### 2.2.5 PPI network construction and core targets screening

To retrieve target protein interactions from “*H. sapiens*,” the cross-targets were entered into the STRING database (https://cn.string-db.org/). The data was analyzed using Cytoscape 3.7.0, and topology parameters like degree, closeness, betweenness centrality, and centrality were calculated using CentiScape 2.2 plugin. Targets above three thresholds were identified using “Filter.” A protein-protein interaction (PPI) network of core targets was constructed using” Networkanalyzer”, with node size and color representing degree values. The nine targets with the highest degree values in the network graph are identified as core targets.

#### 2.2.6 Construction of the “ingredient-pathway-target” network

The “ingredient-pathway-target” network was created in Cytoscape 3.9.1 using the formula, cross-targets, active ingredients, pathways with *p* < 0.01, and top 5 count values. Degree values were calculated using the Centiscape 2.2 plugin, and the top 5 ingredients were chosen as the main active ingredients.

### 2.3 Molecular docking

Core target proteins and main active ingredients were molecularly docked. The 2D structures of the 5 main active ingredients were downloaded in mol2 format from the TCMSP database (https://tcmsp-e.com/, accessed in December 2023). The 3D PDB structures of the nine core targets were downloaded from the RCSB PDB PRO database (https://www.rcsb.org/), AlphaFold Protein Structure Database (https://alphafold.com/), and SWISS-MODEL database (https://swissmodel.expasy.org/, December 2023 query). The core target proteins were prepared using AutoDockTools 1.5.6 software by removing water, adding hydrogens, calculating Gasteiger charges, designating them as receptors, and saving as PDBQT. The main active ingredients were processed by adding hydrogens, setting ligands, detecting torsions, choosing torsions, and saving as PDBQT. AutoDock Vina 1.1.2 software was utilized for docking to produce a set of data with binding energy as the primary parameter. This data was utilized to evaluate the binding properties between the ingredients and the target site. The results with low binding energy were visualized in PyMOL (Version 2.6.0a0 Open-Source).

## 3 Results

### 3.1 Frequency analysis

The initial screening yielded 1,045 documents, 85 documents were finally selected according to the inclusion as well as exclusion criteria, 89 prescriptions were screened by removing duplicate ones, with 151 drugs having 907 occurrences. According to the Ministry of Health and Welfare and the Journal of China Food Drug Administration, there are 110 types of drugs that are now classified as MFH. Based on the MFH list, prescription screening found 38 MFH drugs with 266 occurrences. In Table 2, there were 15 high-frequency MFHs (>4 times) with a total frequency of 227, representing 25% of all medicine frequencies and 85.3% of all MFHs frequencies. They were: Angelicae Sinensis Radix, Astragali Radix, Poria, Persicae Semen, Allii Macrostemonis Bulbus, Ginseng Radix Et Rhizoma, Glycyrrhizae Radix Et Rhizoma, Codonopsis Radix, Puerariae Lobatae Radix, Platycodonis Radix, Crataegi Fructus, Ostreae Concha, Ziziphi Spinosae Semen, Jujubae Fructus, and Amomi Fructus.

**TABLE 2:** Medicine food homology materials appearing over 4 times in prescriptions of MVA.

MFHs	Frequency	Propotion (%)
Angelicae Sinensis Radix (Danggui)	38	4.19
Astragali radix (Huangqi)	29	3.20
Poria (Fuling)	27	2.98
Persicae Semen (Taoren)	19	2.09
Allii Macrostemonis Bulbus (Xiebai)	19	2.09
Ginseng Radix Et Rhizoma (Renshen)	18	1.98
Glycyrrhizae Radix Et Rhizoma (Gancao)	17	1.87
Codonopsis Radix (Dangshen)	14	1.54
Puerariae Lobatae Radix (Gegen)	8	0.88
Platycodonis Radix (Jiegen)	8	0.88
Crataegi Fructus (Shanzha)	8	0.88
Ostreae Concha (Muli)	6	0.66
Ziziphi Spinosae Semen (Suanzaoren)	6	0.66
Jujubae Fructus (Dazao)	5	0.55
Amomi Fructus (Sharen)	5	0.55

### 3.2 Association analysis and network display

We obtained three association rules (Table 3), and all the lift values for these rules are larger than 1, indicating their effectiveness as commonly used combinations of MFH drugs. Among them, the rule {Angelicae Sinensis Radix} => {Persicae Semen} exhibits the highest support and confidence, with 22.89% and 73.68%, respectively. The rule {Persicae Semen} => {Glycyrrhizae Radix Et Rhizoma, Angelicae Sinensis Radix} shows the highest degree of lift, reaching 3.18. Further visualization using a network graph involves setting drug nodes’ size and color intensity based on “degree” to highlight their significance in the network. The thickness and color intensity of connections between drugs are determined using “combine core” to indicate their closeness.

**TABLE 3:** Association rules of MFHs for MVA.

Former item	Latter item	Support/%	Confidence/%	Lift
Angelicae Sinensis Radix (Danggui)	Persicae Semen (Taoren)	22.89	73.68	1.61
Persicae Semen (Taoren)	Glycyrrhizae Radix Et Rhizoma (Gancao) and Angelicae Sinensis Radix (Danggui)	13.25	72.73	3.18
Astragali radix (Huangqi)	Codonopsis Radix (Dangshen)	16.87	71.43	2.04

MFHs: medicinal food homology drugs; MVA: microvascular angina; Support: how freguently the combinations occur in the dataset; Confidence: the reliability of the asociation rule; Lift: the ratio of observed support to the targeted one when the two items are independent. A rule with the lift value >1 implies that these two occurrences are dependent on one another.

**FIGURE 2:**
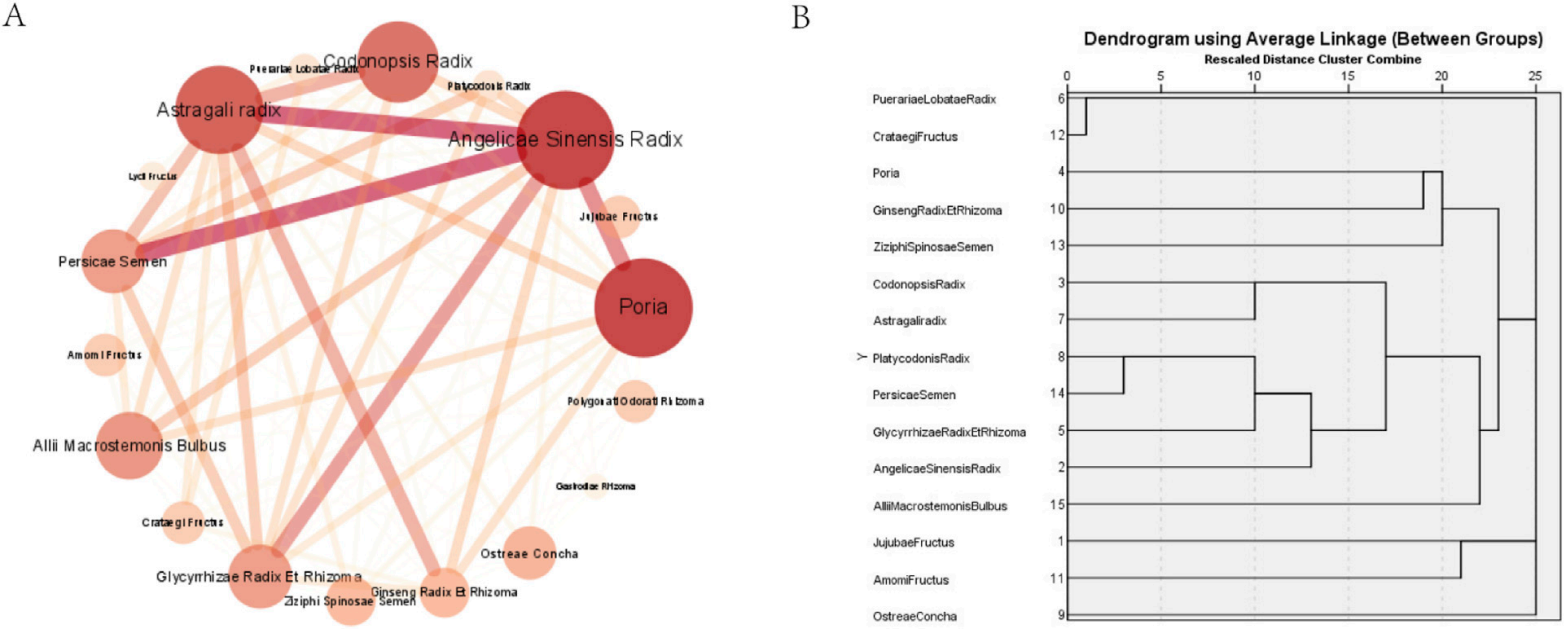
Data analysis **(A)** Drug association degree nelwork diagram. The darker the color and larger the diameter of a drug node, the more critical the drug is. The thicker and darker the edges between the nodes, the stronger the association between the two drugs **(B)** Dendrogram from cluster analysis of medicine food homology drugs for microvascular angina.

According to Table 4 and Figure 2A, Poria, Angelicae Sinensis Radix, Astragali radix, Codonopsis Radix, Allii Macrostemonis Bulbus, Glycyrrhizae Radix Et Rhizoma, and Persicae Semen exhibit the highest centrality in the network, suggesting their crucial role as essential MFH drugs in treating microvascular angina.

**TABLE 4:** Measure nodes by their network features to infer their importance in the network.

MFHs	Network		
	BetweennessCentrality	ClosenessCentrality	Degree
Poria (Fuling)	0.238	0.787	27
Angelicae Sinensis Radix (Danggui)	0.171	0.787	27
Astragali radix (Huangqi)	0.090	0.740	24
Codonopsis Radix (Dangshen)	0.082	0.712	22
Allii Macrostemonis Bulbus (Xiebai)	0.077	0.661	18
Glycyrrhizae Radix Et Rhizoma (Gancao)	0.037	0.649	17
Persicae Semen (Taoren)	0.050	0.649	17
Ostreae Concha (Muli)	0.033	0.617	14
Ginseng Radix Et Rhizoma (Renshen)	0.018	0.607	13
Ziziphi Spinosae Semen (Suanzaoren)	0.018	0.607	13
Crataegi Fructus (Shanzha)	0.008	0.587	11
Jujubae Fructus (Dazao)	0.024	0.587	11
Amomi Fructus (Sharen)	0.010	0.587	11
Polygonati Odorati Rhizoma (Yuzhu)	0.009	0.587	11
Puerariae Lobatae Radix (Gegen)	0.002	0.552	8
Platycodonis Radix (Jiegeng)	0.002	0.536	8
Lycii Fructus (Gouqizi)	0.001	0.544	7
Gastrodiae Rhizoma (Tianma)	0.000	0.514	6

MFHs: medicinal food homology drugs; Degree: the number of network edges between a node and others; Betweenness centrality: counts the network’s shortest node paths; Closeness centrality: The reciprocal of the average distance between nodes. High levels of these three indicators imply node importance.

### 3.3 Hierarchical cluster analysis

Using IBM SPSS Statistics 25, a hierarchical cluster analysis of high-frequency MFHs was carried out, and a cluster analysis dendrogram was produced (Figure 2B). Five new clusters were found using the Euclidean distance of 22.5 (Table 5). Cluster 1: Puerariae Lobatae Radix (Gegen), Crataegi Fructus (Shanzha). Cluster 2: Poria (Fuling), Ginseng Radix Et Rhizoma (Renshen), Ziziphi Spinosae Semen (Suanzaoren). Cluster 3: Codonopsis Radix (Dangshen), Astragali radix (Huangqi), Platycodonis Radix (Jiegeng), Persicae Semen (Taoren), Glycyrrhizae Radix Et Rhizoma (Gancao), Angelicae Sinensis Radix (Danggui), Allii Macrostemonis Bulbus (Xiebai). Cluster 4: Jujubae Fructus (Dazao), Amomi Fructus (Sharen). Cluster 5: Ostreae Concha (Muli).

**TABLE 5:** Cluster analysis.

Cluster	Drug combination
C1	Puerariae Lobatae Radix, Crataegi Fructus
C2	Poria, Ginseng Radix Et Rhizoma, Ziziphi Spinosae Semen
C3	Codonopsis Radix, Astragali radix, Platycodonis Radix, Persicae Semen, Glycyrrhizae Radix Et Rhizoma, Angelicae Sinensis Radix, Allii Macrostemonis Bulbus
C4	Jujubae Fructus, Amomi Fructus
C5	Ostreae Concha

### 3.4 Generation of core MFH formula

The results of frequency analysis, complex network analysis, association analysis, and systematic clustering analysis were comprehensively analyzed to screen out the core MFH drugs and generate a new formula. The drug composition of this new formula is Persicae Semen (Taoren), Angelicae Sinensis Radix (Danggui), and Allii Macrostemonis Bulbus (Xiebai), Codonopsis Radix (Dangshen), Astragali Radix (Huangqi), Platycodonis Radix (Jiegeng), Glycyrrhizae Radix Et Rhizoma (Gancao).

### 3.5 Network pharmacology analysis

#### 3.5.1 Identification of active ingredients and drug targets

Based on the screening criterion, 2, 26, 2, 13, 3, 2, and 27 active ingredients from Persicae Semen (Taoren), Angelicae Sinensis Radix (Danggui), and Allii Macrostemonis Bulbus (Xiebai), Codonopsis Radix (Dangshen), Astragali Radix (Huangqi), Platycodonis Radix (Jiegeng), and Glycyrrhizae Radix Et Rhizoma (Gancao) were obtained from the, ETCM database, respectively. After screening and removing duplicates, 529 drug targets were identified.

#### 3.5.2 Identification of disease targets and cross-targets

In GeneCards, OMIM, and Disgenet databases, 1721, 25, and 39 disease-related targets were obtained, respectively, and after screening and deletion of duplicates, a total of 901 MVA-related targets were identified.

**FIGURE 3:**
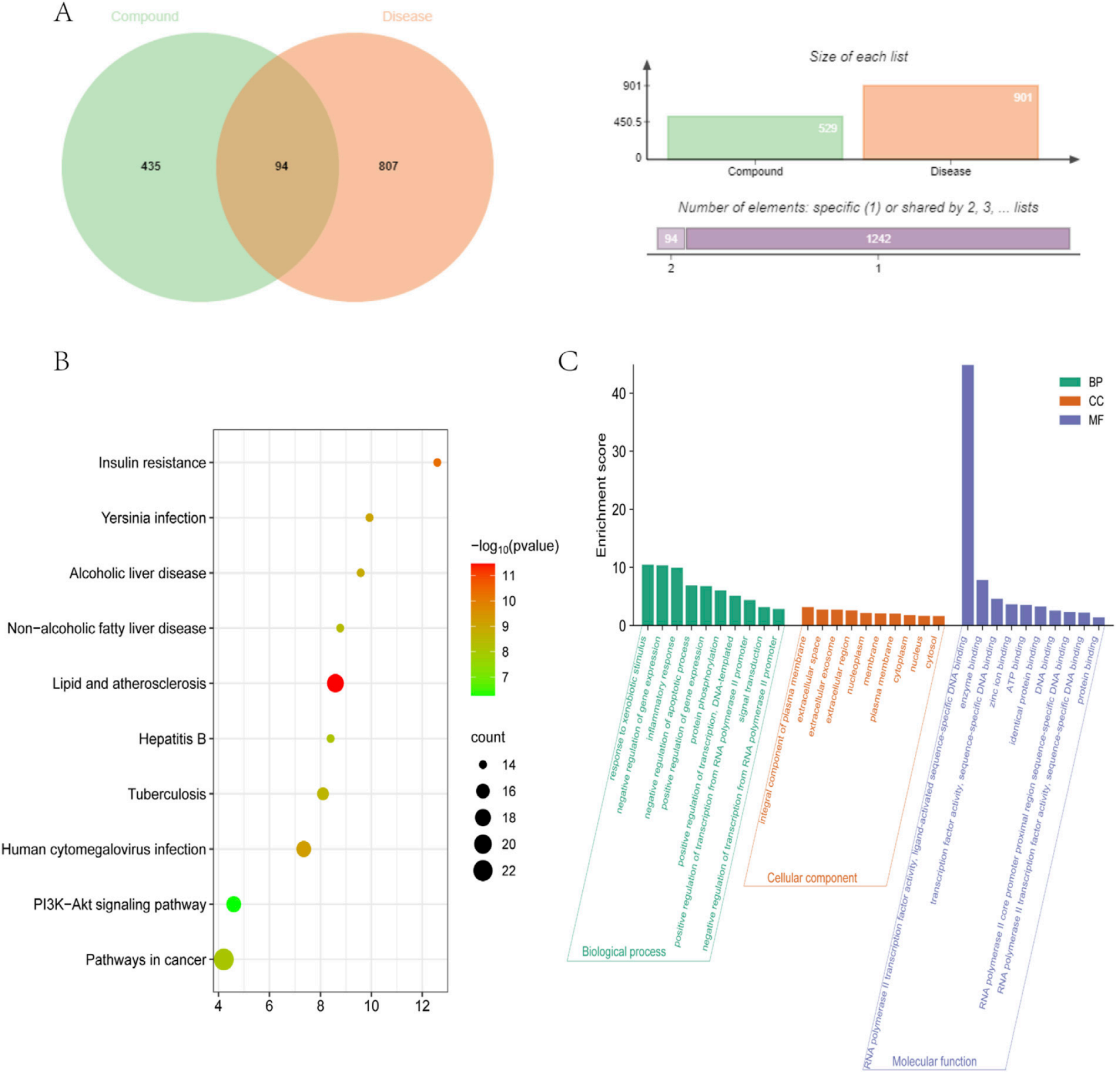
Cross targets and enrichment analysis **(A)** Common genes between disease and compound **(B)** GO function analysis **(C)** KEGG pathways enrichment analysis.

Venn analysis of the above drug targets and disease targets yielded 94 cross-targets, which are considered potential targets of MFH drugs against MVA (Figure 3A).

#### 3.5.3 GO function and KEGG pathways enrichment analysis

To investigate the functions and pathways of 94 cross targets, GO and KEGG enrichment analyses were conducted using the DAVID database.

A total of 799 enriched GO terms were identified (*p* < 0.01), comprising 108 MF terms, 82 CC terms, and 464 BP terms. BP was distributed in response to xenobiotic stimulus, negative regulation of gene expression, inflammatory response, and negative regulation of apoptotic process. CC was mainly involved in integral component of plasma membrane, extracellular space, extracellular exosome, and extracellular region. MF primarily involves RNA polymerase II transcription factor activity, ligand-activated sequence-specific DNA binding, enzyme binding, transcription factor activity, sequence-specific DNA binding, and zinc ion binding. The results are depicted in Figure 3B. The results confirmed that the active components of the MFHF can act as therapeutic agents on MVA through various gene product functions.

A total of 121 KEGG pathways were obtained (*p* < 0.01). The KEGG pathways were mainly enriched in pathways in cancer, lipid and atherosclerosis, human cytomegalovirus infection, and PI3K-Akt signaling pathway. The results show that active components in the MFHF can act as anti-MVA through various signal pathways. We identified and visualized the top 10 pathways, as shown in Table 6 and Figure 3C.

**TABLE 6:** Results of KEGG enrichment analysis (top 10).

No.	Pathway	Count	P-value	Genes
1	hsa05200:Pathways in cancer	23	9.57E-09	PTGER4, GSK3B, HSP90AA1, NOS2, GSTP1, SLC2A1, PTGS2, ESR1, ESR2, NFKB1, IGF1R, MAPK10, NFKBIA, AR, IL6, PIK3CA, CASP3, ABL1, AKT1, PPARG, JAK1, PPARD, MAPK3
2	hsa05417:Lipid and atherosclerosis	19	3.39E-12	ABCA1, HSPA8, GSK3B, HSP90AA1, IFNB1, VLDLR, TNF, NFKB1, MAPK10, NFKBIA, IL6, PIK3CA, IL1B, CASP3, AKT1, PPARG, TLR4, ABCG1, MAPK3
3	hsa05163:Human cytomegalovirus infection	17	7.17E-10	PTGER4, GSK3B, IFNB1, ITGB3, PTGS2, TNF, NFKB1, NFKBIA, IL6, CREB1, PIK3CA, IL1B, CASP3, PTK2B, AKT1, JAK1, MAPK3
4	hsa04151:PI3K-Akt signaling pathway	17	5.28E-07	GSK3B, HSP90AA1, PRKAA1, IFNB1, ITGB3, PIK3CG, NFKB1, INS, IGF1R, IL6, CREB1, PIK3CA, AKT1, TLR4, JAK1, EPHA2, MAPK3
5	hsa05152:Tuberculosis	15	2.92E-09	NOS2, IFNB1, VDR, ITGB2, TNF, NFKB1, MAPK10, IL6, CREB1, IL1B, CASP3, AKT1, TLR4, JAK1, MAPK3
6	hsa04931:Insulin resistance	14	4.77E-11	SREBF1, GSK3B, PRKAA1, SLC2A1, TNF, NFKB1, INS, MAPK10, NFKBIA, IL6, CREB1, PIK3CA, AKT1, PPARA
7	hsa05135:*Yersinia* infection	14	9.78E-10	GSK3B, IFNB1, TNF, NFKB1, ACTB, MAPK10, NFKBIA, IL6, PIK3CA, IL1B, PTK2B, AKT1, TLR4, MAPK3
8	hsa04936:Alcoholic liver disease	14	1.53E-09	SREBF1, GSK3B, PRKAA1, IFNB1, TNF, NFKB1, MAPK10, NFKBIA, IL6, IL1B, CASP3, AKT1, PPARA, TLR4
9	hsa04932:Non-alcoholic fatty liver disease	14	4.51E-09	SREBF1, GSK3B, PRKAA1, TNF, NFKB1, INS, MAPK10, IL6, PIK3CA, IL1B, CASP3, AKT1, PPARG, PPARA
10	hsa05161:Hepatitis B	14	7.73E-09	IFNB1, TNF, NFKB1, MAPK10, NFKBIA, IL6, CREB1, PIK3CA, CASP3, PTK2B, AKT1, TLR4, JAK1, MAPK3

These findings provide the genetic background of MVA for further network pharmacology research.

#### 3.5.4 PPI network construction and core targets screening

**FIGURE 4:**
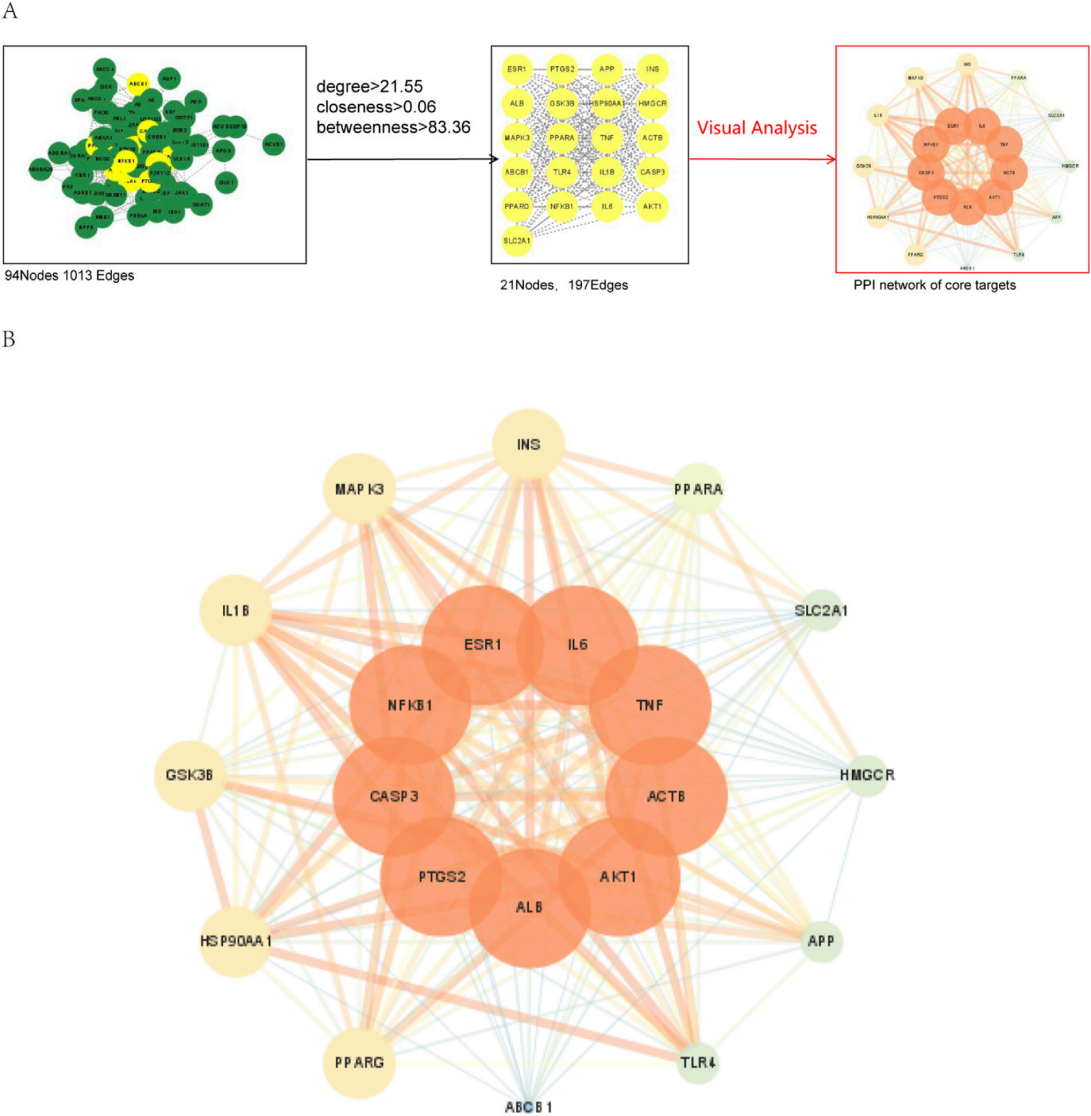
Protein-protein interaction (PPI) network **(A)** The core targets screening process **(B)** Protein-protein interaction (PPI) network of core targets. The width of the line indicates the degree of confidence, the wider the line, the higher the degree of confidence. The core target are circled around the center.

To systematically understand the relationship between targets and identify core targets for subsequent molecular docking, we inputted 94 cross-targets into the STRING database platform. This led to a protein-protein interaction (PPI) network with 94 nodes and 1,013 interaction lines. Nodes represent protein names, and edges represent protein interactions. The initial PPI network was imported into Cytoscape 3.7.0 to visualize the core target network. The average degree value is 21.553, the average CSC value is 0.00583, and the average BC value is 83.362. After filtering based on the average value of the three, a total of 21 nodes and 1,013 edges were obtained. The screening process is depicted in Figure 4A. The nine core targets with the highest degree values were NFKB1, ALB, AKT1, ACTB, TNF, IL6, ESR1, and CASP3, positioned at the center of the network diagram, as shown in Figure 4B.

#### 3.5.5 “ingredient-pathway-target” network

**FIGURE 5:**
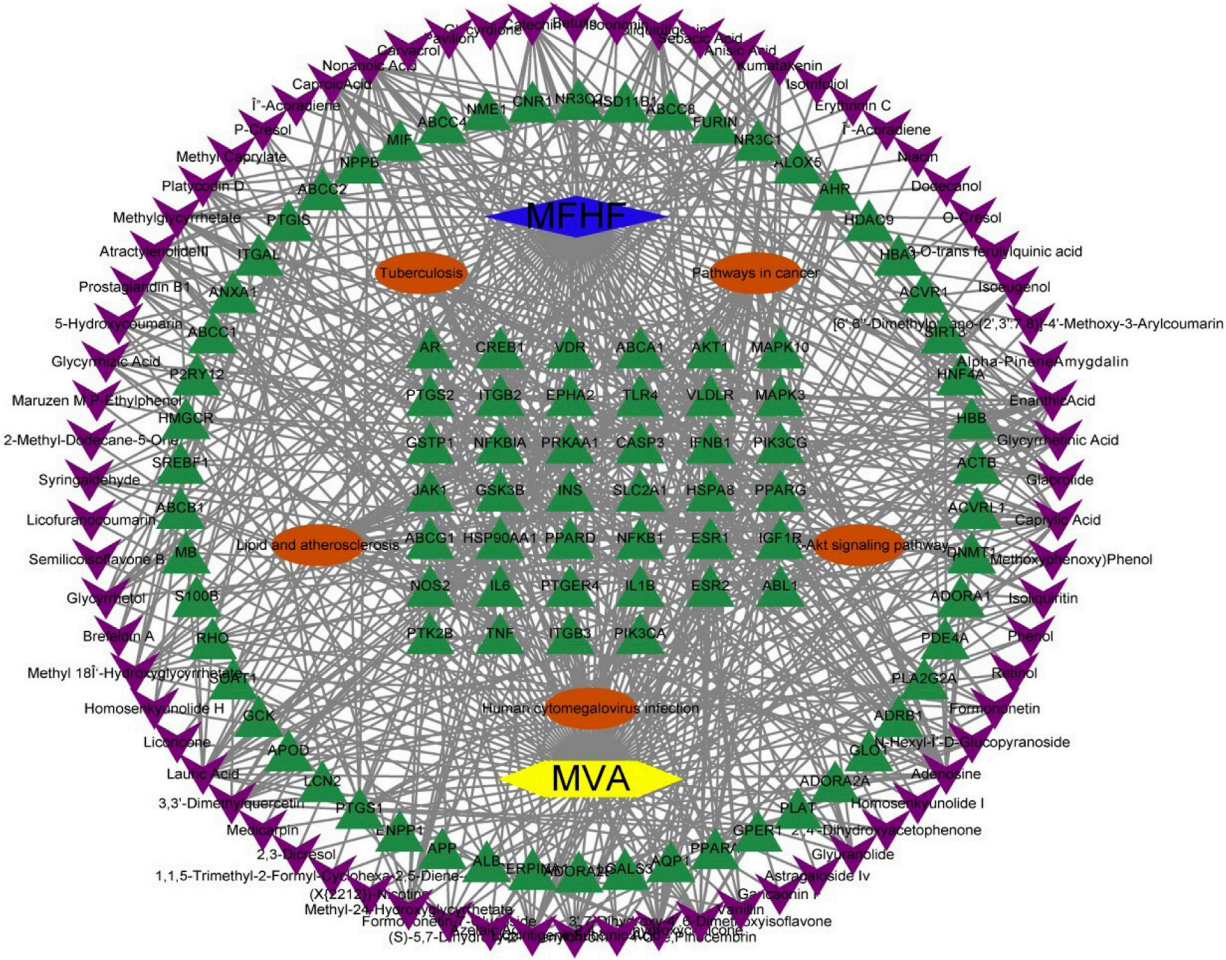
ingredient-target-pathway” network Note: Blue node: medicinal food homology formula; Yellow node: microvascular angina; Red nodes: the first five pathways; Green nodes: targets of the formula for treating the disease.

To gain a deeper insight of the molecular mechanisms behind the targets of MFHF in anti-MVA, we constructed an “ingredient-target-pathway” network (Figure 5). The network consists of 173 nodes and 599 edges. The main active ingredients were selected based on their degree values. Ultimately, we identified Adenosine (degree = 29), Nonanoic Acid (degree = 19), Lauric Acid (degree = 19), Caprylic Acid (degree = 19), and Enanthic Acid (degree = 19). The network diagram suggests that the drug compounds in this MFH formula treat MVA through multiple pathways and targets.

#### 3.5.6 Molecular docking analysis

**FIGURE 6:**
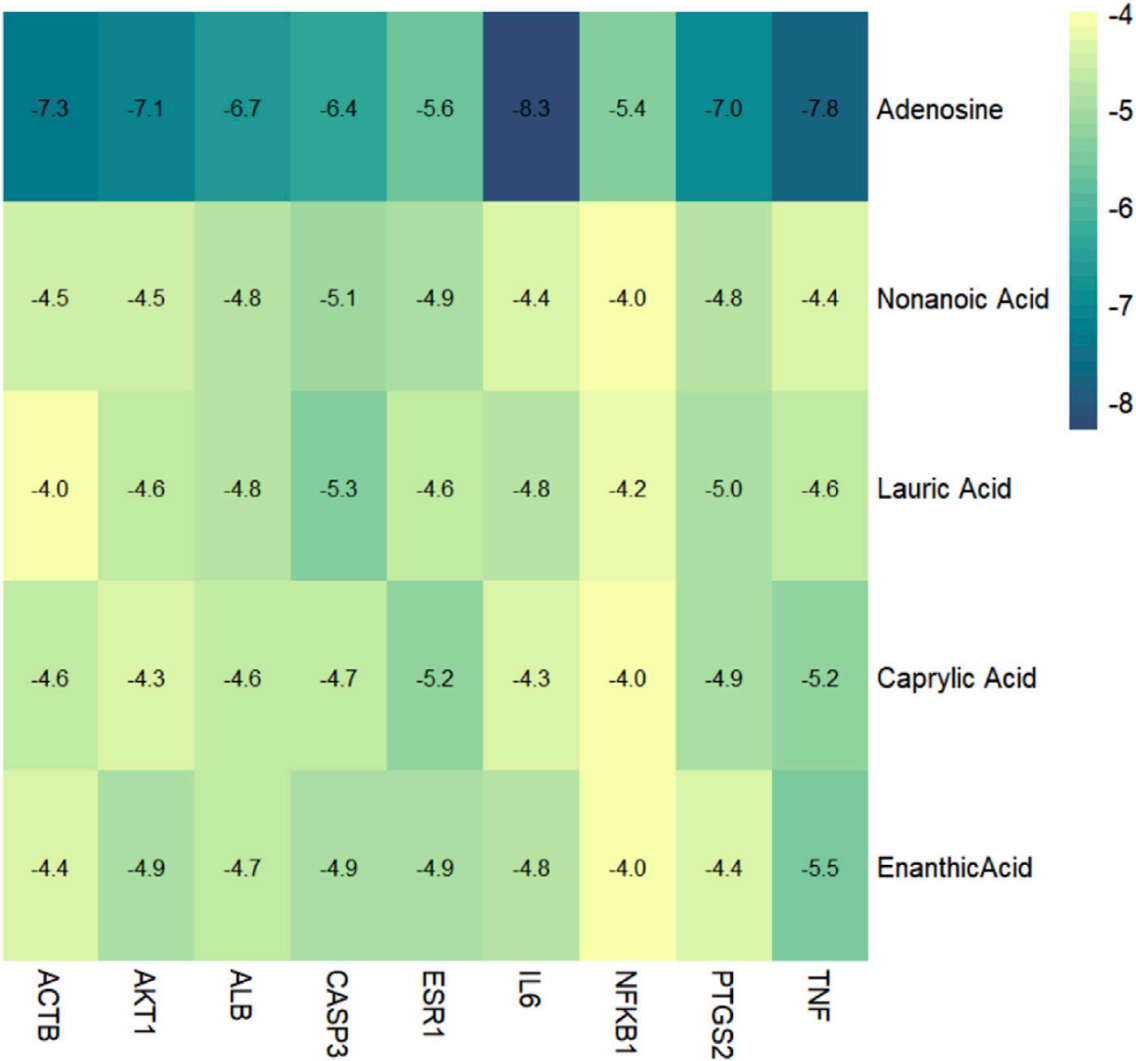
Heatmap of the binding affinity (kcal/mol) of core targets and main compounds.

Through AutoDock Vina 1.1.2 and PyMOL (Version 2.6.0a0 Open-Source), molecular docking was conducted to investigate potential binding modes and evaluate the reliability of interactions between the main active ingredients and core targets. The binding energy results were imported into R 4.3.2, and the pheatmap package was utilized to generate a heatmap visualizing the binding affinity. Darker colors indicate a greater energy release when the ligand and receptor are bound together, suggesting a more stable binding pattern. The binding energy is below 0 kJ/mol, indicating that the ligand and receptor are assumed to be freely bound. The binding energy is below −5 kJ/mol (1.2 kcal/mol), indicating a good affinity 1. Our docking results showed that all binding energies were less than −1.2 kcal/mol (Figure 6). This suggests that the main active ingredients in the study have a good binding capacity with the core targets, confirming the reliability of the results of network pharmacology analysis.

**FIGURE 7:**
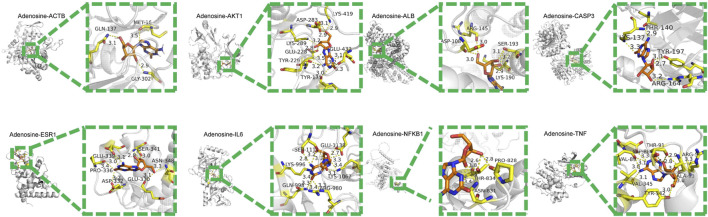
Docking modes of adenosine with core targets Note: The left side of the diagram shows the location of adenosine. The right side of the diagram illustrates the hydrogen bonding of adenosine with core targets. The orange image represents adenosine. The protein targets binding to adenosine are represented by yellow sticks, with the binding sites linked by yellow hydrogen bonds. The length of the hydrogen bonds is indicated next to each bond.

Among all the molecular dockings, adenosine exhibited the lowest binding energies to the core targets, indicating the strongest interactions with the identified core targets. The binding energies were recorded as −7.3, −7.1, −6.7, −6.4, −5.6, −8.3, −5.4, −7.0, and −7.8 kcal/mol (Table 7). This result may be attributed to adenosine’s interaction with amino acid residues of different core targets (Figure 7).

**TABLE 7:** Docking interaction between adenosine and core targets.

Ingredient-target	Key residues	Number of hbonds	Length of hbonds (Å)	Affinity (kcal/mol)	Dist from rmsd l.b	Best mode rmsd u.b
Adenosine-ACTB	GLN-137 MET-16 GLY-302	1 1 1	3.1 3.5 2.9	−7.3	0	0
Adenosine-AKT1	LYS-419 ASP-283 LYS-289 GLU-228 TYR-229 GLU-432 TYR-175	1 2 1 1 2 3 1	2.9 3.1, 2.9 3.2 2.8 3.2, 3.5 3.1, 3.2, 3.3 3.0	−7.1	0	0
Adenosine-ALB	ASP-108 ARG-145 SER-193 LYS-190	1 1 2 1	3.0 3.0 3.1, 3.2 2.9	−6.7	0	0
Adenosine-CASP3	THR-140 LYS-137 TYR-197 ARG-164	1 1 2 1	2.9 3.3 3.0, 2.7 3.2	−6.4	0	0
Adenosine-ESR1	GLU-339 PRO-336 ASP-332 SER-341 ASN-348 GLU-330	2 1 1 2 1 1	3.1, 3.0 3.4 3.2 2.8, 3.0 3.3 3.1	−5.6	0	0
Adenosine-IL6	GLN-998 ARG-980 LYS-1067 GLU-1138 SER-1111 LYS-996	1 2 2 2 3 1	3.3 3.3, 3.4 3.1, 3.4 2.7 2.8, 3.2, 3.1 3.4	−8.3	0	0
Adenosine-NFKB1	PRO-828 THR-834 ASN-831	1 2 2	2.8 2.6, 1.8 3.5, 3.4	−5.4	0	0
Adenosine-TNF	THR-91 ASP-88 VAL-89 VAL-345 TYR-342 ALA-93 ARG-5	3 1 2 1 1 2 1	3.6, 2.8, 2.9 3.3 3.1, 3.0 3.1 3.0 3.0, 2.9 3.3	−7.8	0	0

Furthermore, IL6 demonstrated the lowest binding energy to adenosine at −8.3 kcal/mol and formed multiple hydrogen bonds with amino acid residues GLN-998, ARG-980, LYS-1067, GLU-1138, SER-1111, and LYS-996.

## 4 Disccusion

Patients with MVA caused by CMVD experience major adverse cardiac events Berry et al. (2022). MVA has difficulties in controlling symptoms and a higher rate of subsequent emergency department visits, hospital admissions, or invasive investigations (Aribas et al., 2022). Risk factors for MVA are hypertension, diabetes, insulin resistance, aging, atherosclerosis (AS), hypercholesterolemia, obesity, and others (Herscovici et al., 2018). Studies have shown that coronary microvascular structural or functional dysfunction, inflammatory response, vascular endothelial dysfunction, silent atherosclerosis, and neurological abnormalities with a low threshold for chest pain play important roles in the etiology of the disease (Recio-Mayoral et al., 2013; Berry et al., 2022; Mehta et al., 2022; Soleymani et al., 2022). In coronary microvascular functional dysfunction, the endothelium-dependent and non-endothelium-dependent mechanisms have been demonstrated. Endothelium-dependent mechanisms include impaired vasodilatory responses, vasodilator-vasoconstrictor imbalance (reduced NO release and elevated plasma ET-1 levels, etc.), and an increased contractile response to endothelin and thrombus-mediated contraction. Non-endothelium-dependent mechanisms include the presence of risk factors, estrogen deficiency, and inflammatory response. Structural abnormalities include microvascular remodelling (Soleymani et al., 2022). In addition, there are mechanisms such as increased membrane sodium-hydrogen exchanger activity, platelet aggregation, psychological morbidity, and genetic variants that have been implicated in the development of MVA (Soleymani et al., 2022).

In view of the current lack of efficacy and side effects of western medicines in the treatment of microvascular angina, a core MFH formula was generated in this study, which takes into account the advantages of TCM compound prescription and the food. The MFHF can be widely used as healthcare medicines and healthcare food in our daily lives. It is likely that MFH formula can help people with MVA to relieve anxiety and depression, in the name of “food”.

In Chinese medicine, according to its clinical characteristics of MVA, it is called “true cardiac pain” and “chest bi-impediment/cardiac pain”. “Deficiency of qi and yin” and “intertwined phlegm and blood stasis” are its basic pathogenesis (Zhong et al., 2020). The elements of TCM patterns for MVA in descending order are Blood stasis > Qi deficiency > Turbid phlegm > Qi impediment > Yin deficiency > Pathogenic dampness.

Through data mining, we created a MFHF. The seven constituent drugs of MFHF target the respective pattern element of MVA. Persicae Semen and Angelicae Sinensis Radix circulate blood and transform stasis, Astragali Radix and Codonopsis Radix tonify qi and generate fluid, Platycodonis Radix and Allii Macrostemonis Bulbus circulate qi and transform phlegm, Glycyrrhizae Radix Et Rhizoma relieves spasm and pain, coordinating the rest of ingredients in a formula.

Persicae Semen (PS), also known as Taoren, is the seed of Prunus persica (L.) BATSCH (Rosaceae)(Fukuda et al., 2003). It is commonly used in Asian countries to treat blood stagnation syndromes due to its anticoagulant, antiphlogistic, and anodyne (Xi et al., 2013). MVA patients may have abnormal central autonomic nervous system network (CAN) function. (Cattaneo et al., 2020). PS and its active compounds affect neuron differentiation, which is essential for the CAN to function in the nervous system. Therefore, it is hypothesized that PS regulates CAN by influencing neuron differentiation and serves as a therapeutic agent for MVA. Amygdalin, the active ingredient in PS, can decrease blood viscosity and improve microcirculation to treat CMD associated with MVA (Zhang et al., 2018). Myocardial scarring was prevalent in women with MVA, but PS could prevent scar formation (Xi et al., 2013; Aldiwani et al., 2021). Besides, PS inhibits nuclear factor-kappa B (NF-κB) and tumor necrosis factor-α (TNF-α), which are mediators of endothelial dysfunction (Perrot-Applanat et al., 2011; Xi et al., 2013).

Angelicae Sinensis Radix (ASR), also known as Danggui, is the root of Angelica sinensis (Oliv.) Diels (Apiaceae), widely marketed as a health food and dietary supplement in both Asia and Western countries (Chen et al., 2013). It helps circulate blood, alleviate pain, and tonify qi and blood, exhibiting various pharmacological effects like neuroprotective, immunoregulatory, antioxidant, and hematopoietic activities (Chen et al., 2013).

Astragali Radix (AR), also known as Huangqi, is the root of Angelica sinensis (Oliv.) Diels (Umbelliferae), commonly used to tonify qi and blood, with therapeutic effects on viral and bacterial infections, inflammation, fatigue, and particularly cardiovascular diseases (CVDs) (Chen Z. et al., 2020; Zhang et al., 2020; Li M. et al., 2022). Huangqi extracts, like astragaloside IV, demonstrate strong protective effects against CVDs (Zhang et al., 2020). They can treat AS by inhibiting inflammation, circulating monocytes (MCs) adhesion, lipid disorders, and foam cell formation, exhibiting significant protective effects on vascular endothelial damage (Liu et al., 2022). The mechanism involves diverse pathways, primarily anti-inflammatory and growth factor signaling (Su et al., 2021).

Codonopsis Radix (CR), commonly known as Dangshen, is the root of Codonopsis pilosula (Franch.) Nannf., having effects on tonifying qi, nourishing blood, and promoting bodily fluid production (Luan et al., 2021). The extracts of CR have various pharmacological effects, such as neuroprotection, immune system regulation, anti-tumor properties, endocrine regulation, hypoglycemic effects, improved hematopoietic function, cardiovascular protection, anti-aging, antioxidant, anti-inflammatory, anti-fatigue, and antiviral effects (Dong et al., 2023).

Platycodonis Radix (PR), also known as Jiegeng, is the root of Platycodon grandiflorum (Li Q. et al., 2022). Platycodin D (PD), the main active ingredient, exhibits multiple bioactivities, including analgesic, fat-reducing, insulin resistance improvement, anti-tumor, immune regulation, anti-inflammatory, lipid reduction (Li Q. et al., 2022). PD can prevent infection-induced CMD by blocking the pathway to novel coronavirus infection and exhibits a wide range of pharmacological effects on the cardiovascular system, such as protecting vascular endothelial cells, anti-platelet abnormal activation, anti-atherosclerosis, and so on (Li Q. et al., 2022).

Allii Macrostemonis Bulbus (AMB), also known as Xiebai, bulb of Allium macrosemonis Bulbus and Allium chinense, is considered a “top grade” herb for the treatment of “chest bi-impediment/cardiac pain”, and has the function of activating Yang and removing stasis, circulating qi and resolving stagnation (Yao et al., 2016; Wu et al., 2023). AMB plays a protective role against CVDs. It has various pharmacological activities such as antiplatelet aggregation, hypolipidemic, anti-atherosclerotic, anti-hypertensive, vascular endothelial cell protection, anti-tumor, antispasmodic, antimicrobial, antioxidant, and antidepressant effects (Yao et al., 2016).

Glycyrrhizae Radix et Rhizoma (GRR), also named Gancao and licorice, is the root and rhizome of the Glycyrrhiza uralensis Fisch., Glycyrrhiza inflata Bat., and Glycyrrhizaglabra L (Ding et al., 2022). It has anti-inflammatory, antioxidant, antidiabetic, anti-allergic, antiviral, and immunomodulatory functions, with multiple pharmacological effects on the respiratory, cardiovascular, and immune systems (Li et al., 2020; Ding et al., 2022; Zhang et al., 2024). Besides, GRR may treat AS by inhibiting platelet aggregation, thrombosis and abnormal VSMC proliferation (Liu et al., 2022).

The pharmacological effects of the seven MFH drugs mentioned above form the basis for treating MVA.

Based on the “ingredient-pathway-target” network, five main ingredients (adenosine, nonanoic acid, lauric acid, caprylic acid, and enanthic acid) were identified for their potential roles in preventing and treating MVA by modulating multiple signaling pathways. Adenosine, the main active ingredient in AMB, blocks the synthesis of thromboxane A2 (TXA2) and significantly inhibits platelet aggregation, which causes an alteration in the resting activity of the sodium-hydrogen exchanger in patients with MVA (Applegate and Sane, 2001; Yao et al., 2016). Adenosine possesses analgesic, anti-inflammatory, cardioprotective, antisclerotic, and antifibrotic properties, along with platelet inhibitory and vasodilatory effects (D'Amario et al., 2020). Phosphorylated adenosine forms are involved in cellular energy transfer and regulate various pathologies linked to ischemic heart injury.

Nonanoic acid, lauric acid, caprylic acid, and enanthic acid belong to medium-chain fatty acids (MCFAs), which may play a therapeutic role against MVA by inhibiting platelet aggregation (Takachi et al., 2004). MCFAs also promote fatty acid metabolism, increasing energy production and protecting cardiomyocytes from apoptosis during ischemic heart disease. Estrogen is essential for endothelial integrity and anti-inflammatory properties. MCFAs can help prevent and treat MVA by increasing serum estrogen levels (Zeng et al., 2022). Meanwhile, caprylic acid has potential applications in mitigating oxidative stress, inflammation, apoptosis, stabilizing mitochondria, anti-aging, anti-infective, and improving the circulatory system (Zeng et al., 2022). Overall, MCFAs have potential therapeutic benefits in treating MVA and improving overall health.

Go enrichment analyses reveal that BP primarily involves response to xenobiotic stimulus, CC is related to the integral component of plasma membrane, and MF is primarily associated with ligand-activated sequence-specific DNA binding RNA polymerase II transcription factor activity. Response to xenobiotic stimulus alters cell or organism activity, such as movement, secretion, enzyme production, or gene expression. Exposure to xenobiotics triggers an inflammatory response, contributing to endothelial activation and dysfunction in MVA (Auerbach et al., 2022). The integral component of plasma membrane consists of gene products and protein complexes that maintain ionic gradients associated with elevated Na+/H+ exchange in MVA (Ronquist and Waldenström, 2003).

KEGG analysis indicates that MVA-associated signaling pathways mainly include pathways in cancer, lipid and atherosclerosis, human cytomegalovirus infection, and PI3K-Akt signaling pathway.

Cancer pathways involve PI3K/AKT/mTOR and Ras/MAPK (Yip and Papa, 2021). Inhibiting these pathways reduces inflammation and apoptosis, resulting in cardiovascular protection (Wang et al., 2017). The TNF signaling pathway is involved in the systemic inflammatory response and interacts with MAPK and NF-kappa B pathways, leading to disease progression (Li H. et al., 2022). Gancao, Jiegeng, Huangqi, and Xiebai block these pathways, acting as anti-inflammatory, antioxidant, anti-inflammatory, and anti-platelet aggregation agents and reducing endothelial dysfunction, foam cell formation, and vascular smooth muscle cell proliferation (Yao et al., 2016; Ding et al., 2022; Wu et al., 2023).

Atherosclerosis is a chronic inflammatory disease caused by lipid-rich plaques in arterial blood vessels. The lipid and atherosclerosis pathway is involved in inflammation, foam cell formation, LOX-1-mediated oxLDL uptake, apoptosis, plaque instability, and interacts with other pathways, leading to the progression of MVA (Pirillo et al., 2013).

Human cytomegalovirus (HCMV) is a double-stranded DNA virus that activates PDGFRA and PI3-K/AKT pathways, influencing the host immune system (Yu et al., 2023). It can induce endothelial injury, promote smooth muscle cell mutations, and increase polymorphonuclear leukocyte adhesion, leading to plaque formation and atherosclerosis (Nieto et al., 1996). HCMV infection may also increase the risk of restenosis after conventional coronary balloon angioplasty by inhibiting P53 function (Manegold et al., 1999).

The PI3K-Akt signaling pathway is activated by many types of cellular stimuli or toxic insults and regulates essential cellular functions such as transcription, translation, proliferation, growth, and survival (Dunn and Connor, 2012). The PI3K/Akt/eNOS pathway regulates the phosphorylation of eNOS and modulates downstream pathways to regulate apoptosis and relax coronary microvessels (Chen R. et al., 2020).

NFKB1, ALB, AKT1, ACTB, TNF, IL6, ESR1, CASP3, and PTGS are the core targets in the PPI network. These genes may be very important in preventing and treating MVA. NF-kappa B, a transcription factor, regulates genes involved in immunity, inflammation, and cell survival (Romzova et al., 2006). It is associated with an inflammatory response, atherosclerosis, and endothelial dysfunction, affecting disease progression in MVA (Fiordelisi et al., 2019). The ALB gene encodes albumin, which possesses anti-inflammatory, antioxidant, anticoagulant, and antiplatelet aggregation activities, so hypoalbuminemia is an independent prognosticator of many cardiovascular diseases, including ischemic heart disease (Chien et al., 2017). AKT1 is relevant to the development of MVA due to its role in energy homeostasis, obesity, and brown fat organogenesis (Sanchez-Gurmaches et al., 2019). ACTB encodes β-actin, which is involved in vascular remodeling and contributes to cardiovascular disease (Yang et al., 2020). TNF induces intracellular signal pathways, inflammation, and immunity. IL-6 levels may be considered a diagnostic marker for MVA (Gurzău et al., 2021). Estrogen receptor alpha (ESR1) inhibits NF-κB signaling, decreasing inflammation-associated cytokines and activating nitric oxide synthase (NOS3) and endothelial NO production (Wu et al., 2022). Antidepressants have been proposed for treatment, with CASP3 gene expression positively correlated with depression duration and the number of episodes (Bliźniewska-Kowalska et al., 2023).

Nevertheless, our current study has certain limitations. The definition of MVA is vague, and there are no clear evidence-based diagnostic guidelines. Therefore, the homogeneity of MVA diagnoses in different literature retrieved cannot be guaranteed. In addition, the molecular docking technique still has some limitations, and the efficacy and safety of this MFHF should be rigorously verified by randomized controlled trials or systematic evaluations in the future.

## 5 Conclusion

MVA remains challenging, and understanding how to prevent this disease through diet is crucial. However, No previous studies have been conducted on the MFH for MVA. Our study introduces a novel multi-ingredient, multi-target, and multi-pathway MFHF to treat MVA, comprising PR, ASR, AMB, CR, AR, PR, and GRR. Leveraging network pharmacology and molecular docking, we identified key MFHF components targeting TCM patterns for MVA. These components, widely used in health foods and supplements with minimal toxicity, target core proteins (NFKB1, ALB, AKT1, ACTB, TNF, IL6, ESR1, CASP3, and PTGS) to modulate cancer, lipid and atherosclerosis, human cytomegalovirus infection, and the PI3K-Akt signaling pathway. Our findings suggest MFHF’s pleiotropic effects on MVA, including antioxidative, anti-inflammatory, analgesic, antiplatelet, vasodilatory, endothelial protective, and cardioprotective actions. This study provides insights into CMVD management, bridging ancient wisdom with modern science for healthcare, disease prevention, and natural therapies, thereby offering potential edible medication applications.

## Data Availability

The raw data supporting the conclusions of this article will be made available by the authors, without undue reservation.
